# Pair Interaction of Catalytical Sphere Dimers in Chemically Active Media

**DOI:** 10.3390/mi9010035

**Published:** 2018-01-17

**Authors:** Jing-Min Shi, Ru-Fei Cui, Jie Xiao, Li-Yan Qiao, Jun-Wen Mao, Jiang-Xing Chen

**Affiliations:** 1 Department of Physics, Hangzhou Dianzi University, Hangzhou 310018, China; 161070042@hdu.edu.cn (J.-M.S.); 151070020@hdu.edu.cn (J.X.); 2 Department of Physics, Zhejiang University, Hangzhou 310027, China; 21636030@zju.edu.cn; 3 Department of Physics, Huzhou University, Huzhou 313000, China; jwmao@zjhu.edu.cn

**Keywords:** pair interaction, catalytically sphere dimer, chemically active medium, multiparticle collision dynamics, phase diagram

## Abstract

We study the pair dynamics of two self-propelled sphere dimers in the chemically active medium in which a cubic autocatalytic chemical reaction takes place. Concentration gradient around the dimer, created by reactions occurring on the catalytic sphere surface and responsible for the self-propulsion, is greatly influenced by the chemical activities of the environment. Consequently, the pair dynamics of two dimers mediated by the concentration field are affected. In the particle-based mesoscopic simulation, we combine molecular dynamics (MD) for potential interactions and reactive multiparticle collision dynamics (RMPC) for solvent flow and bulk reactions. Our results indicate three different configurations between a pair of dimers after the collision, i.e., two possible scenarios of bound dimer pairs and one unbound dimer pair. A phase diagram is sketched as a function of the rate coefficients of the environment reactions. Since the pair interactions are the basic elements of larger scale systems, we believe the results may shed light on the understanding of the collective dynamics.

## 1. Introduction

Collective dynamics of active particles have attracted great attention in the interdisciplinary fields including biochemistry, materials, and physics in the past decade [1,2,3,4]. From the fundamental point, the study on this subject can help us to give insight into the far from equilibrium physics underlying the collective behavior of biological entities. The dynamic of a motor in a many-motor system behaves differently from that of an isolated motor [5,6]. In collections of such motors, the presence of other motors in the surroundings leads to mutual interaction, which would not only change the motion of a single particle but also lead to the emergence of cooperative phenomena [7,8,9].

From principle individual particle motion to complex multiparticle behavior, the pair interactions set up a bridge as the basic elements of larger scale systems. Thus, studies about pair dynamics are essential for further understanding of collective behavior [10,11]. A great number of interesting phenomena in two or more hydrodynamical collective motors has been observed [12,13]. Since the interactions among individual particles become important, it is common to observe phenomena like clustering and rectification effects [14,15,16]. In a recent example, the bimetallic rods or dimers would form pairs transiently due to the specific short-range interactions [17]. Wykes et al. found this kind of rods could interact with each other and pair up to form a swimmer or a rotor which exhibit the fundamental form of motility: translation and rotation [18]. Thakur et al. found a variety of bound and unbound states after undergoing a collision of dimer pair [19].

Biochemical environments are generally in and out of equilibrium state [20,21]. Microswimmers often perform tasks in complex environments which are chemically active [22]. The environmental reactions may change the nonequilibrium concentration gradients that are a central element of the propulsion mechanism. A natural raised interesting question is how the nonequilibrium state affects the interaction between a pair of motors. Obviously, the dynamics in such media will be quite different. For example, the periodic supply of fuel to a motor as a result of autonomous oscillations in the surrounding medium will cause oscillations in the motor velocity [23,24,25]. In this article, we investigate the dynamics of two chemically powered sphere dimer motors in a chemically active medium. The chemical dimer motors are immersed into a solution full of fuel particles that also take part in the bulk phase reactions. The concentration gradient around the dimer, created by reactions occurring on the catalytic sphere surface and responsible for the self-propulsion, are affected by the bulk environmental reactions. Consequently, the communications between the dimer pair are altered, which results in different configurations of dimer pair. In terms of the particles-based method combined with molecular dynamics (MD) and reactive multiparticle collision dynamics (RMPC), we can adjust the activities of the environment to study the pair dynamics of sphere dimers.

## 2. Mesoscopic Model and Simulation Method

The self-propelled nanodimers consist of the catalytic (*C*) and noncatalytic (*N*) spheres which are linked by a fixed distance *R*. The dimers are surrounded with the point-like fluid (solvent) particles comprising *A* and *B* species. In our system, we have considered two sphere-dimer motors. The catalytic sphere catalyzes the irreversible chemical reaction by converting *A* (fuel particles) to *B* (product particles) when *A* encounters *C*.

(1) $$\begin{array}{c} A+C\to B+C,\;\mathrm{on}\;\mathrm{the}\;\mathrm{dimer}\;\mathrm{motor} \end{array}$$

This model mimics generic features of synthetic nanorods: catalytic reactions occur on one end and the reaction product interacts differently with the catalytic and noncatalytic ends. The motors based on self-diffusiophoresis had been employed to represent the dynamics of sphere dimers comprising a non-catalytic silica sphere connected to a catalytic platinum sphere in experiment [26].

Also there is autocatalytic reaction occurring in the environments with rate constants $k_{1}$ and $k_{2}$ [19]

(2) $$\begin{array}{c} B+2A\overset{k_{1}}{\underset{k_{2}}{⇌}}3A,\;\mathrm{in}\;\mathrm{the}\;\mathrm{environment} \end{array}$$

The iodate/arsenous acid system can be accurately modeled by such cubic autocatalysis [27]. Here, we select such a special reaction to construct the complex environment. The reactant or product in Equation (1) is then involved in the cubic autocatalytic reaction in Equation (2) taking place in the bulk phase environment, which forms a reaction network coupled by diffusion. The A and B species interact with the dimer sphere through repulsive Lennard-Jones (LJ) interactions of the form

(3) $$V_{\alpha S}=4\epsilon_{\alpha S}\left[{\left(\sigma_{S}/r\right)}^{12}-{\left(\sigma_{S}/r\right)}^{6}+\frac{1}{4}\right]\theta \left(r_{c}-r\right)$$

where θ(r) is the Heaviside function and $r_{c}=2^{1/6}\sigma_{S}$ is the cutoff distance. The notation $V_{\alpha S}$, where S=C,N and α=A,B, are used to denote various interactions between solvent and dimer monomers. We take $V_{AC}=V_{BC}=V_{AN}$, which are characterized by the same energy parameter $\epsilon_{A}$. However, interactions between the *N* sphere and *B* molecules, $V_{BN}$, have a different energy parameter $\epsilon_{B}$ and a distance parameter $\sigma_{N}$. The asymmetric potentials are responsible for the diffusiophoretic mechanism of the self-propulsion. The interaction between these two different sphere dimers, denoted by subscripts 1 and 2, respectively, is also described by the repulsive LJ potentials Equation (3) with an energy parameter $\epsilon_{D}$ and a cutoff distance $r_{c}=2^{1/6}\sigma_{D}$ where $\sigma_{D}=\sigma_{S_{1}}+\sigma_{S_{2}}+\delta_{D}$ with $\delta_{D}=0.2$.

The time evolution of the entire system is carried out using a hybrid MD-RMPC scheme, which combines molecular dynamics (MD) for sphere dimers and reactive multiparticle collision (RMPC) dynamics for the fluid particles [28,29,30]. The hybrid MD-RMPC dynamics consists of the free streaming step and a collision step. In the streaming step, the system including dimers and solvent molecules are propagated by Newton’s equations of motion, which are described by molecular dynamics (MD) through deriving forces from Equation (3) with time interval $\Delta t_{MD}$.

There is no net force among solvent particles. Instead, the interaction between the solvent particles is described by multiparticle collisions dynamics (MPC). In the collision step, the system is divided into cubic cells with size a=1 and rotation operators ${\hat{\omega }}_{\alpha }$ which are assigned to each cell from some set of rotation operators. After the center-of-mass velocity ${\vec{v}}_{cm}$ of each cell ξ is calculated from ${\vec{v}}_{cm}=\sum_{j=1}^{N_{c}}{\vec{v}}_{j}/N_{c}$, where $N_{c}$ is the total number of particles in the cell, the post-collision velocity ${\vec{v}}_{i}\left(t+\tau \right)$ of each particle *i* within the same cell can be obtained according to the rotation rule

(4) $${\vec{v}}_{i}\left(t+\tau \right)={\vec{v}}_{cm}\left(t\right)+{\hat{\omega }}_{\alpha }\left({\vec{v}}_{i}\left(t\right)-{\vec{v}}_{cm}\left(t\right)\right)$$

Grid shifting was employed to ensure Galilean invariance. The hybrid MD-MPC dynamics includes fluctuations, conserves mass, momentum and energy, and accounts for coupling between the *C* sphere motion and fluid flows.

The cubic autocatalytic reactions in the bulk phase take place independently in each cell at each MPC collision step $\Delta t_{MPC}$ [28]. In the cell ξ, the bulk reactions in Equation (2) occur according the following probabilities: the forward reaction with probability $\frac{a_{1}}{a_{0}}\left(1-e^{-a_{0}\Delta t_{MPC}}\right)$; the backward reaction with probability $\frac{a_{2}}{a_{0}}\left(1-e^{-a_{0}\Delta t_{MPC}}\right)$; and no reaction with probability $e^{-a_{0}\Delta t_{MPC}}$, where $a_{1}=k_{1}N_{B}N_{A}\left(N_{A}-1\right)$, $a_{2}=k_{2}N_{A}\left(N_{A}-1\right)\left(N_{A}-2\right)$, and $a_{0}=a_{1}+a_{2}$ with $N_{A}$ ($N_{B}$) the total number of *A* (*B*) particles in cell ξ.

In our simulations, all quantities are reported in dimensionless LJ units based on energy ϵ, mass *m* and distance σ parameters: r/σ→r, $t{\left(\epsilon /m\sigma^{2}\right)}^{1/2}\to t$, and $k_{B}T/\epsilon \to T$. The simulation box of the system is divided into 50×50×50 cells, and the average number density in each cell is $n_{0}≃10$. The MPC rotation angle is fixed at $\alpha =90^{\circ }$. The masses for A and B are both fixed at m=1. The system temperature is $k_{B}T=1/6$ and the LJ potential parameters are chosen to be $\epsilon_{A}=1.0$ and $\epsilon_{B}=0.1$. The diameters of the catalytic spheres are $d_{C}=4.0$ while the diameters of the noncatalytic spheres are $d_{N}=8.0$. The MD time step is $\Delta t_{MD}=0.01$ and the time step for MPC is $\Delta t_{MPC}=0.05$. We chose $\epsilon_{D}=1.0$, δ=0.8, and R=6.8.

## 3. Results and Discussions

**Figure 1:**
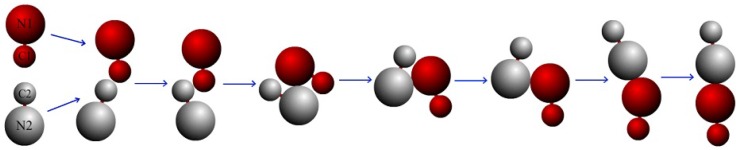
Schematic representation shows the process of formation of a Brownian dimer pair resulted from the collision of two self-propelled nanodimers. The reaction rate coefficient are $k_{1}=0.001$ and $k_{2}=0$.

Two sphere dimers are initially separated by distance 20.0, and they are targeted to undergo collinear collisions (see the first configuration in Figure 1). Since the fluctuation from the solvent particles, two dimers facing to each other from self-propulsion would not always strictly stay on the same line before the collision. When the motors approach each other and collide, two ultimate possible scenarios are observed: either they would (i) interact and then separate with independent motions or (ii) contact and form a bound pair.

Since the interactions of dimer pair are mediated by solvent fields, it is necessary to study the general distribution of species *A* and *B* resulted from chemical reactions to analyze the problem how the pair forms the post-collision configurations. The autocatalytic chemical reaction A+C→B+C converting fuel *A* to product *B*, which takes place whenever *A* particles enter into the boundary layer of the *C* sphere, may create a gradient concentration field of species *A* and *B* around the dimer motors. Since the interaction potentials of species *A* and *B* with the *N* sphere are different, the self-generated chemical gradients of these species give rise to an asymmetric force on the motor directed along its internuclear axis that propels it in solution. In addition, the autocatalytic reaction in the solution, $B+2A\overset{k_{1}}{\underset{k_{2}}{⇌}}3A$ characterized by the intrinsic rates $k_{1}$ and $k_{2}$, greatly influences the gradient fields of *A* and *B*. Therefore, it is evident that the pair configurations are determined by the environment reactions.

Firstly, we discuss the simple case where the bulk reaction is irreversible, i.e., the rate coefficients $k_{2}=0$. An example illustrating the collision process is plotted in Figure 1. In this case, when the pair approaches each other, they collide firstly and then rotate around each other (see the third and fourth configurations). They keep on moving past each other (the fifth and sixth configurations) until they form a bound pair and move together (the seventh and eighth configurations). The bound-pair may reorient and execute Brownian motion, however, it has no self-propulsion velocity. The stable pair configuration, labeled by “Brownian dimer pair (BP)” is described by the characteristics in Table 1. As shown in Figure 1, the pair is almost linear, which is confirmed by the angles $\theta_{1}$ and $\theta_{2}$. One can see the value $\theta_{1}$ is close to $180^{\circ }$ while the value of $\theta_{2}$ is small. The presented distances in Table 1, i.e., $r_{N_{1}N_{2}}$, $r_{C_{1}C_{2}}$, $r_{C_{1}N_{2}}$, and $r_{N_{1}C_{2}}$ all verify the BP configurations.

**Figure 2:**
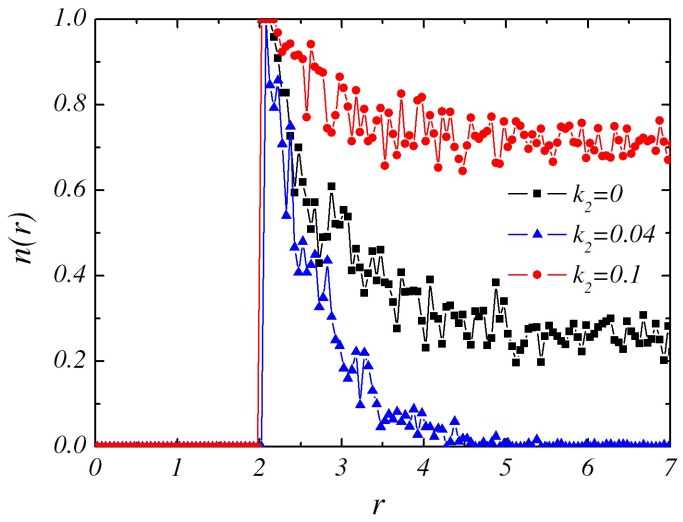
The concentration field of *B* species as a function of distance *r* from the center of the catalytic sphere in the steady state with $k_{1}=0.001$.

It is the gradient field of *B* particles that determines the self-propulsion of individual dimer as well as the pair interactions. Thus, it is essential to study its behavior under the influences of reactions in Equations (1) and (2). The forward reaction in Equation (2) requires both sufficient *A* and *B* particles while the backward reaction needs only *A* particles. Since Equation (1) on *C* sphere surface generates gradient field of *B* particles shown in Figure 2, the probability of forward reaction in Equation (2) is small in the region too close (bare *A* particles) or too far (bare *B* particles) from the *C* sphere. Thus, the forward reaction often occurs in those radial regions, e.g., around *r* = 3–5 in Figure 2 where profiles of radial distribution of *B* particles with different $k_{2}$ are plotted. To the backward reaction, it frequently appears in the bulk solution full of *A* particles and a little farther from *C* sphere. Thus, although Equation (2) is reversible, the forward and backward reactions generally take place in different radial zones. As $k_{2}$ is increased slightly, e.g., from $k_{2}=0$ to $k_{2}=0.04$, the forward reaction still plays a major role and it decreases B particles. Therefore, the concentration gradient is getting steeper.

**Figure 3:**
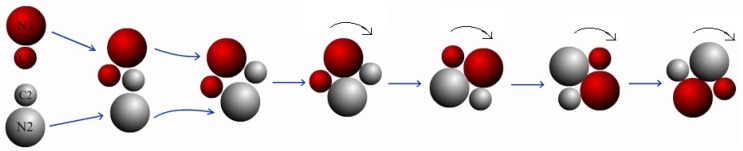
Schematic representation shows the process of formation of a rotating dimer pair resulted from the collision of two self-propelled nanodimers. The reaction rate coefficient are $k_{1}=0.0005$ and $k_{2}=0.02$.

As a consequence of the steeper gradient of *B* particles, a new bound-pair appears. The process is illustrated in Figure 3. Specifically, when the two dimers approach and collide with each other (the first and second configurations), they are strong attracted mutually (the third and fourth configurations), instead of brushing away in the BP case. The reason is that when the $N_{2}$ ($N_{1}$) monomer achieves contact with $C_{1}$ ($C_{2}$), it experiences bigger attraction from the steeper gradient of B particles around $C_{1}$ ($C_{2}$), which prevents the $N_{2}$ moving on. Consequently, a stable configuration is ultimately formed and the bound pair rotates clockwise since the attractive force is pointing from $N_{2}$ to $C_{1}$ (and $N_{1}$ to $C_{2}$), which results in a force moment correspondingly. The last four configurations in Figure 3 depict the rotation of the dimer pair. This stable bound state is labeled as “rotating dimer pair” (RP). Compared to the cases in BP state, the value of $r_{C_{1}C_{2}}$ in Table 1 is quite small since the *C* monomers come into contact and assemble. The values of $r_{C_{1}N_{2}}$ and $r_{C_{2}N_{1}}$ are almost the same here, and they are much smaller than the values in the BP configurations. Since the two *N* monomers keep on touching, the values of $r_{N_{1}N_{2}}$ in BP and RP are approximately equal.

With larger values of $k_{2}=0.1$, the autocatalytic reaction in Equation (2) occurs mainly in a backward direction. The *A* particles produced by the forward reaction are converted back to *B* quickly, producing a flat *B* concentration profile(see Figure 2). So there is very little interaction between these two sphere dimers to keep them bound and hence the pair would move pass each other and “escape” from each other. The state that the two dimers move independently and move away from each other after collision is labeled as “Independent dimer pair” (IP).

Since the two spatiotemporal reactions in Equations (1) and (2) with different response radial regions and diffusions of solvent particles, the dynamics of the system is getting very complex. If $k_{1}$ is very small, e.g., $k_{1}=0.0001$, simulation gives us the transition from RP to IP. However, if $k_{1}$ is substantially increased to $k_{1}=0.0015$, the transition from BP to IP takes place without RP state when $k_{2}$ is increased.

**Figure 4:**
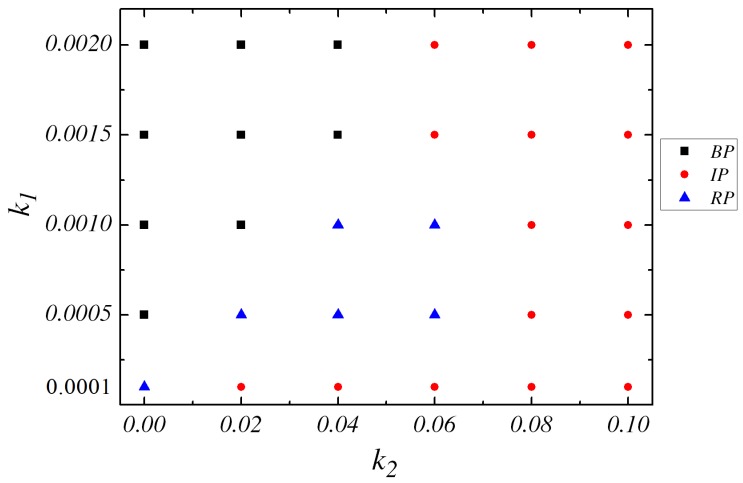
Phase diagram showing the post-collision nature of the dimer pair in the $k_{1}-k_{2}$ plane. Different three regions: two types of bound dimer pairs like Brownian (black square) and rotating dimer pair (blue triangle) and one unbound pair: independently moving dimers (red dot).

**Figure 5:**
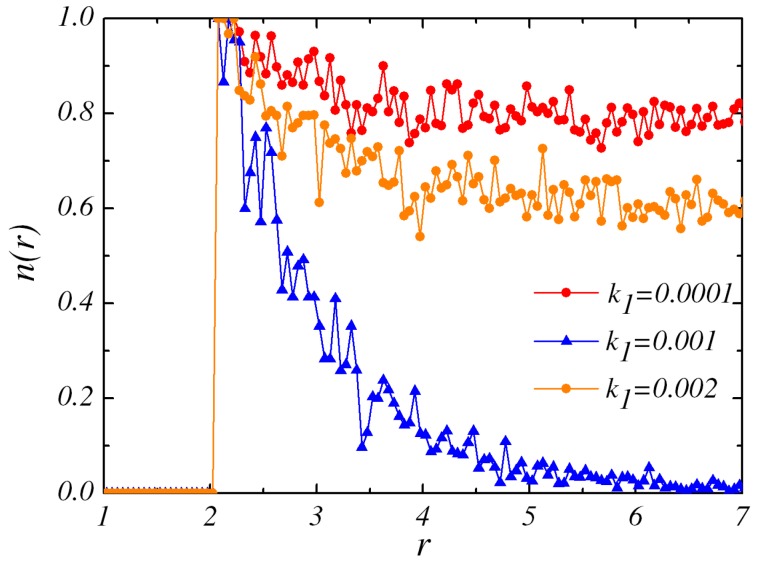
The concentration field of *B* species as a function of distance *r* from the center of the catalytic sphere in the steady state with $k_{2}=0.06$.

Then, we increase the value of $k_{1}$ while $k_{2}=0.06$. If $k_{2}=0$, the RP configuration is observed at very small $k_{1}$ (0.0001). Subsequently, the increase of $k_{1}$ results in the appearance of BP state. If $k_{2}$ is decreased, e.g., $k_{2}=0.02$ and $k_{2}=0.04$, IP, RP, BP successively are obtained when $k_{1}$ is enhanced. Figure 4 demonstrates the steady state concentration field of particle *B* around the catalytic sphere for different values of rate constant $k_{1}$ with $k_{2}=0.06$. When $k_{1}=0.0001$, the bulk conversion *B* to *A* is pretty slow and then most *A* particles in the gradient field would become *B* by the backward reaction, which results in small nonequilibrium *B* concentration gradient around the dimers. An IP state is observed in the phase diagram. At higher $k_{1}$ (0.001), it becomes easier for particles *B* converting to *A* in the solution. Especially it is possible to have this bulk reaction near the *N* sphere and it gives rise to the enhanced decay of density field. The strong concentration field would have the significant effect on the pair interaction of the dimers. The time evolution of the pair shows that they would form RP again. Additionally, those *B* particles produced by the *C* catalytic reaction are converted back to *A* at a fast reaction rate when $k_{1}=0.002$. Less *A* particles could be supplied near the dimer in this situation and then it yields a flat *B* concentration profile. From the discussion above, it is confirmed that the configuration of IP is observed in the first and the third cases. As $k_{2}$ is increased to large value, such as $k_{2}=0.08$, only IP configurations are observed at any $k_{1}$. Different configurations emerge as a result of the concentration gradient of the species in the environment which are influenced by the values of $k_{1}$ and $k_{2}$. The phase diagram which includes all the three final configurations( i.e., BP, RP and IP) is sketched in Figure 5 in the $k_{1}-k_{2}$ plane.

## 4. Conclusions

A coarse grain model has been developed to study the pair interaction of catalytical dimer motor propelled by self-diffusiophoresis. Two dimer motors are placed in a chemically active medium where reactions occur at the catalytic monomers and the reactant or product of this reaction is involved in a cubic autocatalytic reaction taking place in the bulk phase environment. The environmental reactions change the non-equilibrium concentration gradients that are a central element of the propulsion mechanism and the consequent pair dynamics. By altering the chemical activities of the medium through changing the intrinsic rates coefficient, the pair shows rich and complex dynamics and forms two bound states, i.e., a rotating dimer pair and Brownian dimer pair. The underlying mechanisms are discussed. A phase diagram describing the dependence of pair configurations on the chemical activities of the environment is presented. The studies in this paper can be extended to other types of motors and more complicated realistic environmental reactions networks.

## Figures and Tables

**Table 1:** The averaged distance of $r_{N_{1}N_{2}}$, $r_{C_{1}C_{2}}$, $r_{C_{1}N_{2}}$, $r_{N_{1}C_{2}}$ obtained from the bound Brownian dimer pair (BP) and Rotating dimer pair (RP) configurations, respectively. $\theta_{1}=\arccos\left({\hat{r}}_{N_{2}N_{1}}\cdot {\hat{r}}_{C_{1}N_{1}}\right)$, $\theta_{2}=\arccos\left({\hat{r}}_{N_{2}N_{1}}\cdot {\hat{r}}_{C_{2}N_{2}}\right)$, where ${\hat{r}}_{C_{1}N_{1}}$ (or ${\hat{r}}_{C_{2}N_{2}}$) and ${\hat{r}}_{N_{2}N_{1}}$ are the unit vectors pointing from *C* to *N* and from $N_{1}$ to $N_{2}$, respectively.

Configuration	$r_{C_{1}C_{2}}$	$r_{C_{1}N_{2}}$	$r_{N_{1}C_{2}}$	$r_{N_{1}N_{2}}$	$\theta_{1}$	$\theta_{2}$
RP	10.938	6.319	6.315	7.119	53.925	126.096
BP	19.937	13.713	13.761	7.227	156.162	22.231
